# Leucine-rich repeat-containing 56 promotes breast cancer progression via modulation of the RhoA/ROCKs signaling axis

**DOI:** 10.1186/s43556-025-00271-w

**Published:** 2025-05-19

**Authors:** Xiqian Zhou, Jiaxin Wang, Meiling Lu, Lin Fang, Junyong Zhao, Dengfeng Li

**Affiliations:** 1 Department of Breast and Thyroid Surgery,Institute of Breast Disease, Shanghai Tenth People’s Hospital, Tongji University School of Medicine, NO.301 Yanchang Middle Road, Shanghai, 200072 People’s Republic of China; 2 School of Medicine, Tongji University, Shanghai, 200092 China; 3 Department of Central Laboratory, School of Life Sciences and Technology, Shanghai Tenth People’s Hospital of Tongji University, Tongji University, Shanghai, 200072 China

**Keywords:** Leucine-rich repeat-containing 56 (LRRC56), Breast cancer, Progression, Intraflagellar transport 88 (IFT88), RhoA/ROCKs signaling

## Abstract

Breast cancer is one of the most common malignancies with a poor five-year survival rate with metastatic disease among women. It has well been documented that leucine-rich repeat-containing (LRRC) family of proteins are remarkably and aberrantly dysregulated across diverse cancer types. Notably, leucine-rich repeat-containing 56 (LRRC56) was found upregulated in metastatic breast cancer, and plays a crucial role for the movement of cilia via intraflagellar transport 88 (IFT88). However, the role for LRRC56 in breast cancer progression and regulation of IFT88 and associated pathways in metastatic progression of breast cancer has not been defined. Via in vitro functional assessments, we found that LRRC56 pivotally influences the proliferative, migratory and invasive capabilities of cancer cells. Further, via in-vivo assessments, we demonstrated that downregulation of LRRC56 effectively inhibits the growth of breast cancer xenograft tumors and their metastasis to the lungs. Mechanistically, we found that LRRC56 interacts with IFT88 to regulate yes-associated protein 1 (YAP1) expression via modulating the Ras homolog family member A (RhoA)/ Rho-associated protein kinases (ROCKs) signaling pathway. LRRC56 also regulates the expression of integrins and several other key molecules including MMP2, MMP9, FAK, as well as markers of epithelial-mesenchymal transition such as E-cadherin and N-cadherin. In summary, our results demonstrate that overexpression of LRRC56 promotes breast cancer progression via upregulating IFT88/YAP1-RhoA/ROCKs pathway, reprogramming extracellular matrix, and enhancing epithelial-mesenchymal transition. These findings highlight a critical role of LRRC56 in promoting breast cancer progression, suggesting that targeting of LRRC56 may offer a promising strategy for treating metastatic breast cancer.

**Supplementary Information:**

The online version contains supplementary material available at 10.1186/s43556-025-00271-w.

## Introduction

Breast cancer (BC), the most prevalent cancer among women, also ranks second in the global incidence of malignancies [1]. According to the latest statistics, approximately 2.3 million new cases of BC were diagnosed globally in 2022, representing about 11.5% of all new cancer cases [1]. In China, the number of new BC cases is increasing steadily, posing a significant public health concern [2]. The pathogenesis of BC is intricate, encompassing genetic and signaling pathway abnormalities [3]. Among these, metastatic breast cancer (MBC) stands out as a particularly challenging type, being a leading cause of mortality among patients [4]. Early-stage BC patients have a remarkably high chance of cure, with a 5-year survival rate exceeding 95%. However, the prognosis declines substantially once metastasis occurs, with the survival rate dropping to merely 28% [5]. Therefore, understanding the pathogenesis of MBC remains crucial to developing more effective treatments and improving patient outcomes.

To identify novel, targeted treatment strategies, we previously analyzed the ONCOMINE database with using ‘metastatic breast cancer’ as the key word. The analysis revealed that Leucine-rich repeat-containing 56 (LRRC56) exhibits higher expression in the metastatic samples [6]. LRRC56, which belongs to the leucine-rich repeat-containing (LRRC) family, is predicted to reside in cilium [7], and multiple studies have documented its role in facilitating dynamin transport during ciliary and flagellar motility, thereby contributing to flagellar movement in eukaryotic cells [7]. LRRC56 has been identified as a transient cargo within the intraflagellar transport (IFT) sequence, where it contributes to the maturation of outer dynein arms (ODAs), which are critical for the motility of cilia in eukaryotic cells [8]. Furthermore, LRRC56 may influence cell motility through its interaction with intraflagellar transport 88 (IFT88) [7]. Similarly, IFT88 has been shown to play a critical role in cell motility by regulating the yes-associated protein 1 (YAP1) [9]. These findings support the hypothesis that LRRC56 enhances the metastatic potential of BC cells by modulating the function of IFT88. These findings suggest that LRRC56 may promote the metastatic potential of BC through the modulation of IFT88 function. IFT88, in turn, has been shown to regulate cell motility by modulating the activity of Yes-associated protein 1 (YAP1) [9]. These findings suggest that LRRC56 may enhance the metastatic behavior of BC cells by modulating IFT88 function.

The extracellular matrix (ECM) is a vital component of the tumor microenvironment (TME), which comprises a complex macromolecular network of collagens, proteoglycans, glycoproteins, glycosaminoglycans, and enzymes. This network plays a critical role in supporting the biomechanical functions of cells, regulating key cellular processes such as adhesion, proliferation, and intercellular communication [10, 11]. Dysregulation of ECM components is closely associated with tumor progression, metastasis, and resistance to therapy [12, 13]. Alterations in ECM stiffness and mechanical properties significantly impact cell migration, invasion, and the formation of metastatic niches [14–16]. Matrix metalloproteinases (MMPs), the primary enzymes involved in ECM degradation and tissue remodeling, have been shown to facilitate cancer cell proliferation, invasion, epithelial-mesenchymal transition (EMT), metastasis, and angiogenesis [17, 18].Integrin family members, which serve as crucial mediators of cell-ECM interactions, play pivotal roles in cancer progression, metastasis, and therapy resistance, particularly including BC [19, 20]. Integrins facilitate EMT, thereby promoting cancer progression [21]. An increasing body of evidence suggests that integrin-targeted therapies may reduce cancer metastasis [22, 23].

Recent studies have highlighted how ECM remodeling promotes EMT, a critical process in BC metastasis [24, 25]. EMT facilitates interactions between tumor cells and the ECM, thereby facilitating metastasis of cancer cells [26]. EMT plays a pivotal role in BC progression by promoting tumor cell invasion and metastasis [27]. The Ras homolog family member A (RhoA)/Rho-associated protein kinases (ROCKs) signaling pathway plays a critical role in regulating cellular migration, invasion, and tumor progression in BC [28]. RhoA, a small GTPase, activates ROCKs, which subsequently regulate the actin cytoskeleton and cell contractility, leading to alterations in cell shape and motility [29]. Various studies demonstrated that RhoA/ROCK signaling is essential for cancer metastasis, particularly during EMT process [30]. Overactivation of the RhoA/ROCK pathway promotes BC cell motility by modulating actin filaments and focal adhesions, thereby increasing metastatic potential [31]. Moreover, this pathway influences the tumor microenvironment by modulating stromal cell behavior, collagen remodeling, and ECM interactions, thus fostering BC progression [32]. Recent advances have also emphasized the involvement of RhoA/ROCK signaling in regulating breast cancer-associated angiogenesis and lymph angiogenesis, both of which are crucial for tumor growth and metastasis [33].

Despite limited research on LRRC56 in tumor, our work aims to explore its role in BC metastasis. Specifically, we seek to investigate the biological mechanisms underlying its involvement in BC progression and to examine how LRRC56 regulates IFT88 and related molecular mechanisms involved in metastatic processes. Our experimental findings demonstrate that LRRC56 promotes BC progression through interaction with IFT88, subsequently modulating the RhoA/ROCK signaling pathway. These results provide novel mechanistic insights into potential therapeutic strategies for advanced breast cancer.

## Results

### The LRRC56 highly expressed in breast cancer

**Fig. 1:**
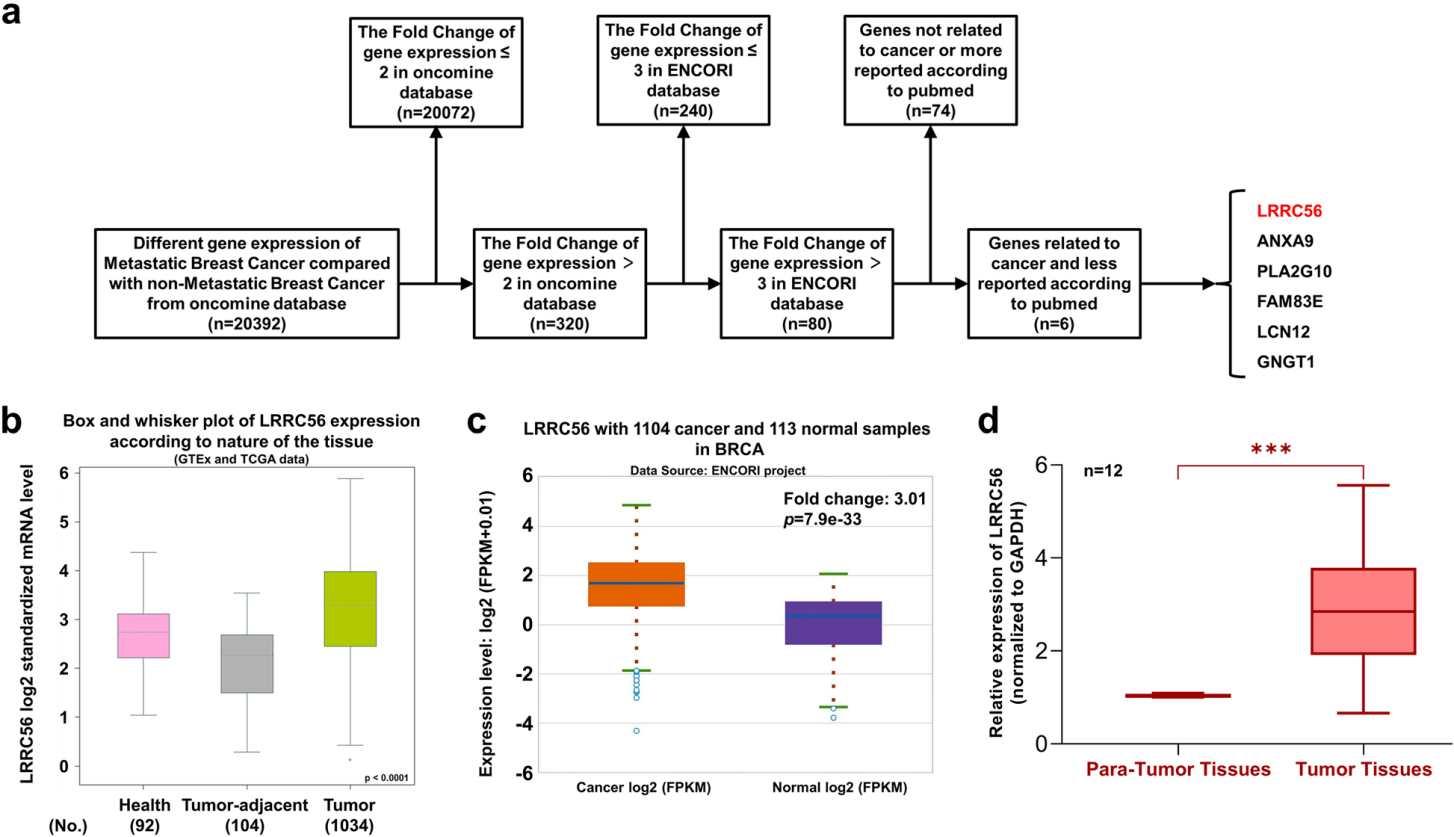
LRRC56 highly expressed in BC. (**a**) Flowchart outlining the selection process for identifying LRRC56 as a candidate gene in BC;(**b**, **c**) LRRC56 expression is significantly upregulated in BC tissues, as analyzed in the bc-GenExMiner and ENCORI databases;(**d**) RT-qPCR validation of LRRC56 overexpression in BC tissues compared to adjacent normal tissues (*n* = 12 per group). *p < 0.05, **p < 0.01, ***p < *0.001*

To identify potential key regulators in MBC, we queried the ONCOMINE database. Notably, a series of differentially expressed genes were identified and gene expression data were subsequently filtered based on fold changes from both the ONCOMINE and ENCORI databases [34], focusing on genes with a fold change greater than three and limited research in cancer (Fig. 1a). Ultimately, we selected LRRC56 as a gene of interest due to its potential involvement in the metastatic process. To investigate LRRC56 expression in BC, we expanded our analysis to additional databases, including bc-GenExMiner [35], and ENCORI. As shown in Fig. 1b-c, the data revealed LRRC56 highly expressed in BC tissues. Furthermore, we validated LRRC56 expression in BC tissues (Fig. 1d), confirming its high expression in tumor samples. Our data indicated that LRRC56 highly expressed in BC and was a potential oncogene in BC progression.

### LRRC56 induces breast cancer cellular progression

**Fig. 2:**
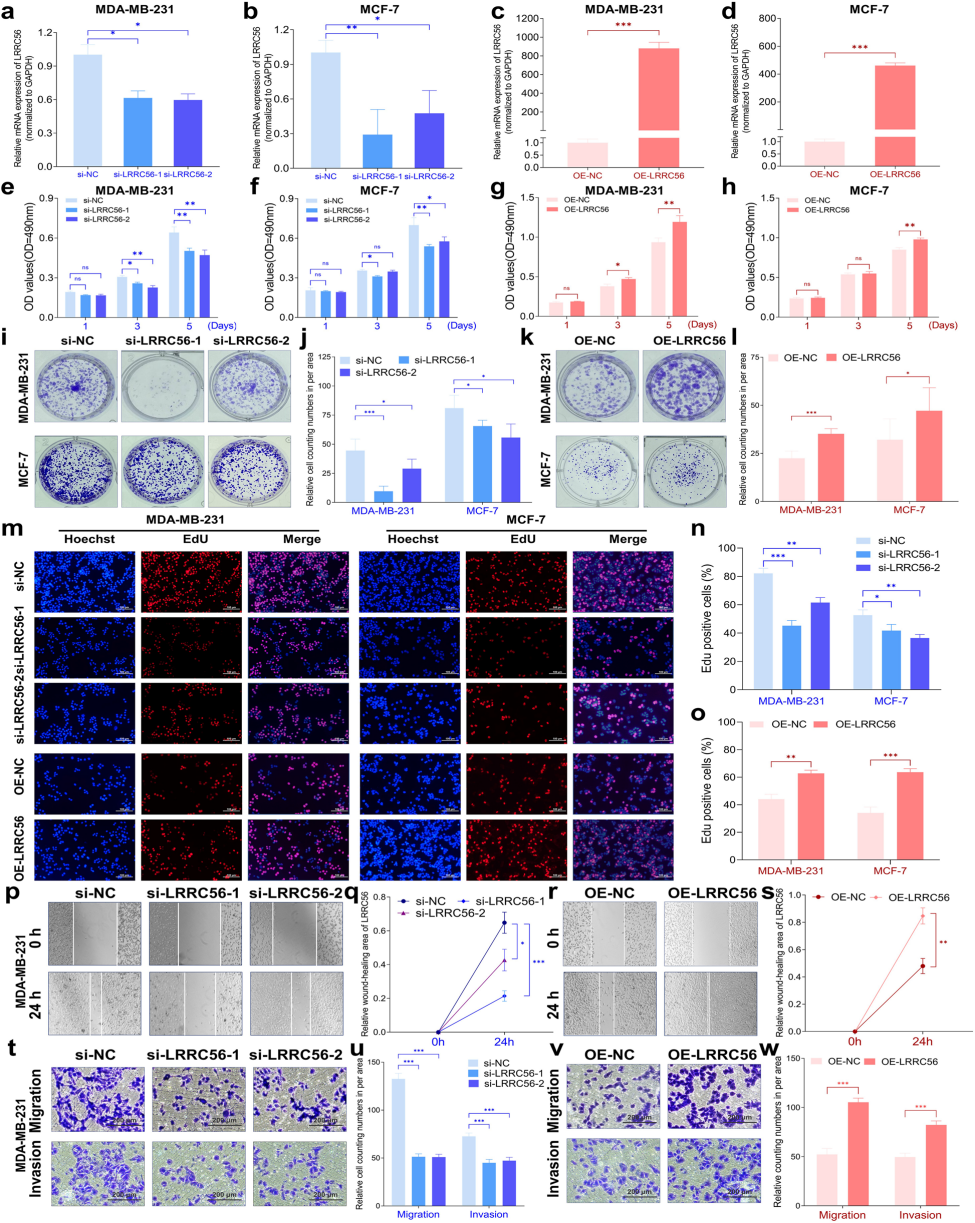
LRRC56 promotes BC progression. (**a **and **b**) si-LRRC56 effectively reduced LRRC56 expression in BC cells; (**c **and **d**) Overexpression plasmid of LRRC56 significantly increased LRRC56 expression in BC cells; (**e **and **f**) Downregulation of LRRC56 inhibited the proliferation of BC cells; (**g **and **h**) Upregulation of LRRC56 promoted the proliferation of BC cells; (**i** and **j**) Reduced LRRC56 expression impaired colony formation ability in BC cells; (**k** and **l**) Enhanced LRRC56 expression promoted colony formation ability in BC cells; (**m**–**o**) EdU assay revealed that LRRC56 upregulation promotes the proliferation of BC cells; (**p** and **q**) Downregulation of LRRC56 inhibited the motility of MDA-MB-231 BC cells; (**r **and **s**) Upregulation of LRRC56 enhanced the motility of MDA-MB-231 BC cells; (**t **and **u**) Downregulation of LRRC56 inhibited migratory and invasive capacities of MDA-MB-231 BC cells; (**v **and **w**) Overexpression of LRRC56 promoted migratory and invasive capacities of MDA-MB-231 BC cells. *p < 0.05, **p < 0.01, ***p < *0.001*

To assess the function of LRRC56 in BC, we altered the expression of LRRC56 in BC cells (Fig. 2a-d). MTT proliferation assays revealed that LRRC56 knockdown significantly inhibited BC cell proliferation, while its overexpression increased cell proliferative capacity (Fig. 2e-h). Colony formation assays further supported these findings, showing that reduced LRRC56 expression decreased the colony formation (Fig. 2i-j), whereas overexpression promoted colony formation (Fig. 2k-l). Additionally, EdU assay incorporation assays indicated that knockdown of LRRC56 suppressed BC cell proliferation, while its overexpression accelerated this process (Fig. 2m-o). Collectively, our findings highlight the that LRRC56 promotes cellular proliferation, underscoring its potential as a key regulator of tumorigenesis in BC.

To further investigate the role of LRRC56 in BC progression, we conducted wound-healing and transwell assays to assess its effects on cell migration and invasion. As shown in Fig. 2p-s, downregulation of LRRC56 significantly reduced the motility of BC cells, while its overexpression enhanced cell motility. The transwell assay further supported these findings, demonstrating that LRRC56 knockdown diminished the motility of MDA-MB-231 cells, whereas its overexpression augmented these abilities (Fig. 2t-w). Taken together, our data indicated that LRRC56 promotes proliferative, migratory and invasive capabilities of BC cells.

### LRRC56 regulates the progression in breast cancer viaRhoA/ROCKs pathway and crosstalk with ECM

**Fig. 3:**
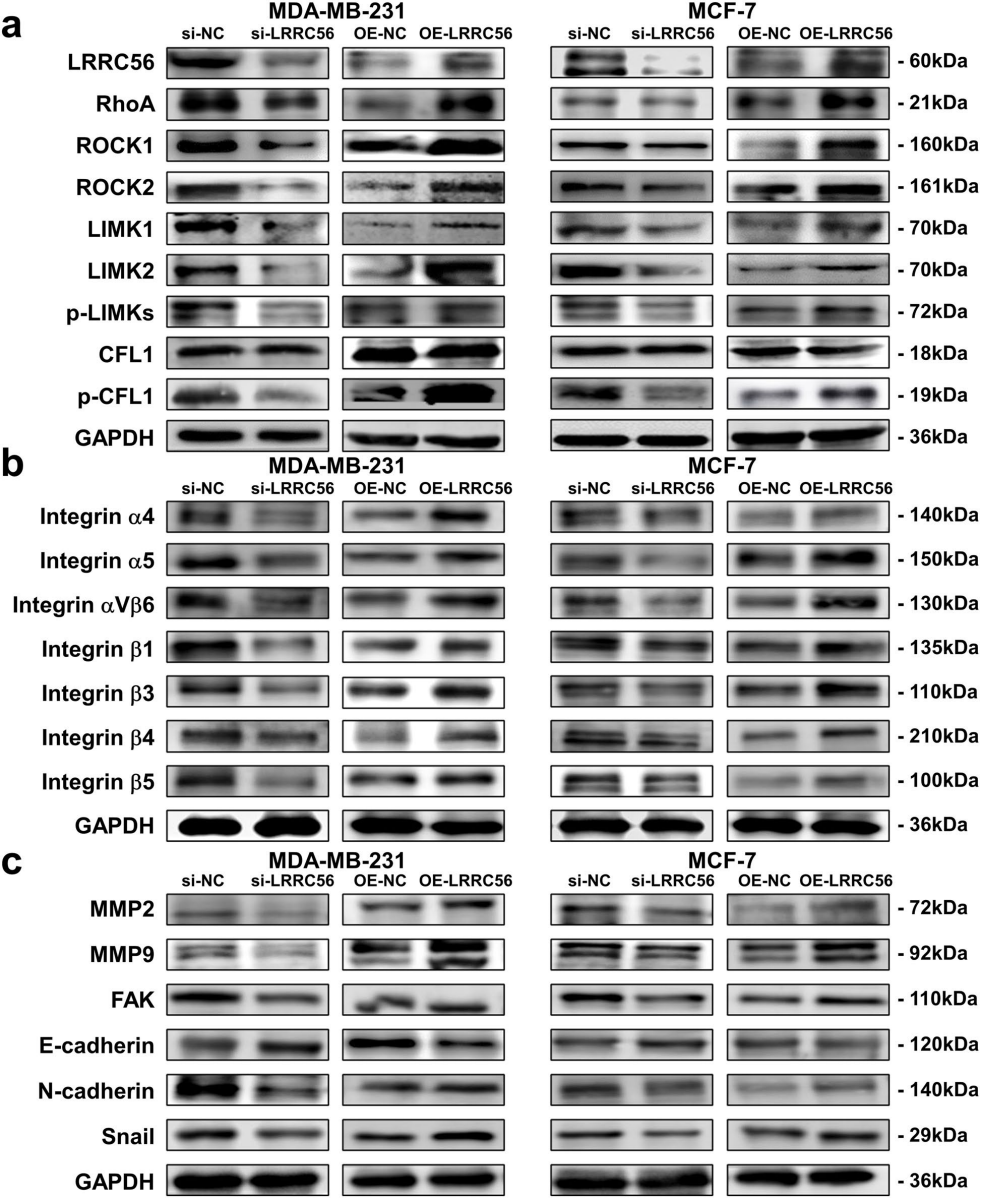
LRRC56 Regulates RhoA/ROCKs Pathway, ECM, and EMT Protein Expression. (**a**) LRRC56 regulates RhoA/ROCKs axis protein expression in MDA-MB-231 and MCF-7 cells; (**b**) LRRC56 regulates integrin protein expression in MDA-MB-231 and MCF-7 cells; (**c**) LRRC56 regulates MMP2, MMP9, FAK, and EMT protein expression in MDA-MB-231 and MCF-7 cells

As the RhoA/ROCKs signaling pathway has been implicated in cell motility, we sought to determine whether LRRC56 modulates this pathway in BC cells, according to our previous findings [36]. The data showed that silencing LRRC56 expression in BC cells decreased protein expression of key RhoA/ROCKs pathway components, while overexpression of LRRC56 increased these protein levels (Fig. 3a, Fig. S1a-d). Given the well-established connection between integrins, ECM, and cell migration, we next investigated the relationship between LRRC56 and integrin family members. In addition, downregulation of LRRC56 reduced cellular expression of integrin proteins, whereas its upregulation enhanced the levels of integrins’ proteins (Fig. 3b, Fig. S1e-h). Since integrins are known to activate signaling pathways by binding to ECM ligands and initiating the EMT, we also examined the link between LRRC56 and EMT-associated proteins. Our findings indicated that downregulation of LRRC56 suppressed the EMT, while its overexpression promoted this process (Fig. 3c, Fig. S1i-l). In summary, LRRC56 appears to be a key regulator in BC progression through its modulation on RhoA/ROCKs pathway and its interaction with the ECM.

### LRRC56 interacts with IFT88 to regulate YAP1 expression

**Fig. 4:**
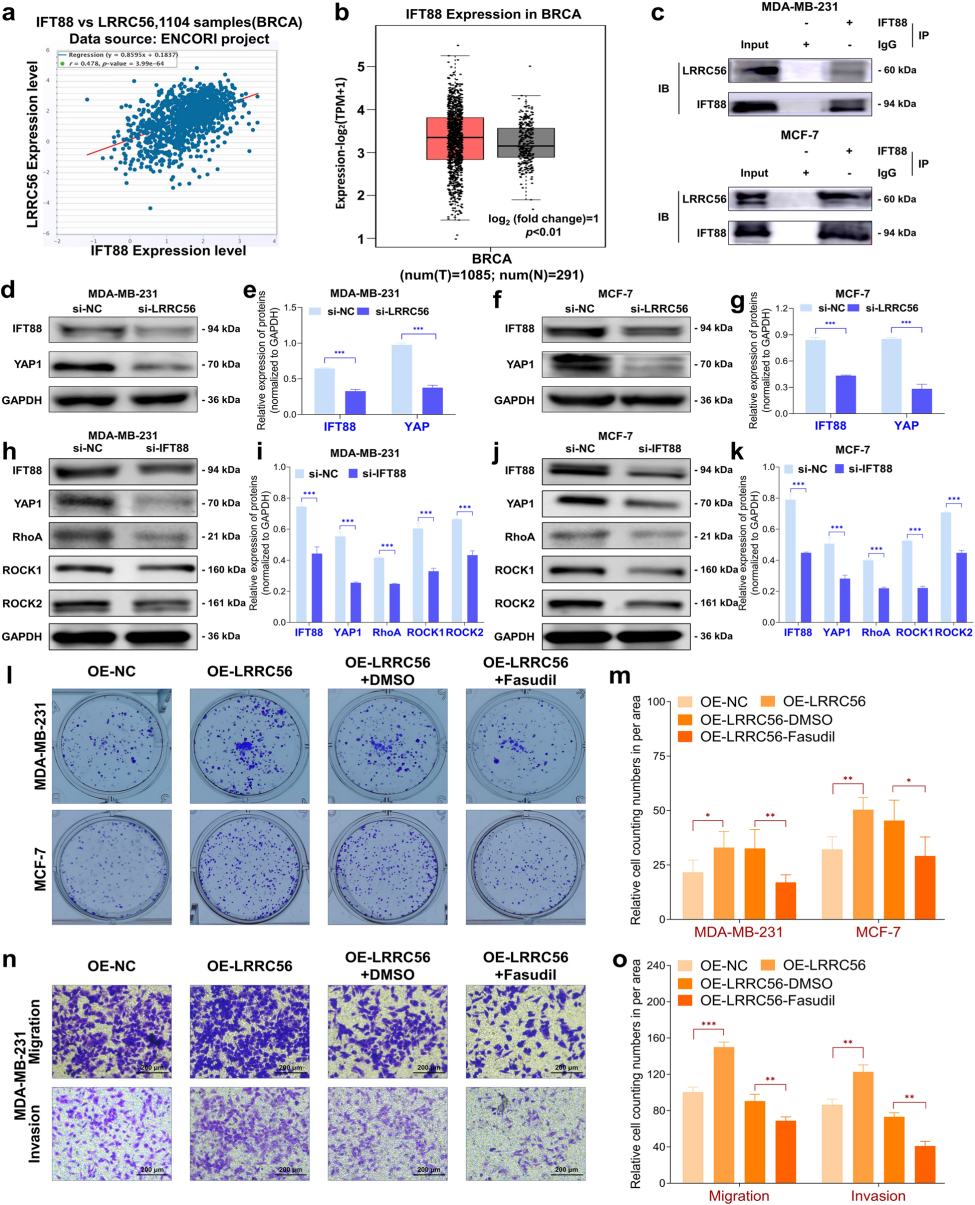
LRRC56 interacts with IFT88 to regulate RhoA/ROCKs pathway in BC. (**a**) LRRC56 is positively correlated with IFT88 in BC; (**b**)IFT88 highly expressed in BC; (**c**) LRRC56 co-interacted with IFT88 in BC cells; (**d **and **e**) Downregulation of IFT88 and YAP1 expression in MDA-MB-231 cells; (**f **and **g**) Downregulation of IFT88 and YAP1 expression in MCF-7 cells; (**h **and **i**) Downregulation of IFT88 reduced YAP1/RhoA/ROCKs protein expression in MDA-MB-231 cells. (**j** and **k**) Downregulation of IFT88 reduced YAP1/RhoA/ROCKs protein expression in MCF-7 cells. (**l** and **m**) Rescue experiments using RhoA/ROCKs inhibitors demonstrate the regulation of colony formation by LRRC56 in BC cells.; (**n** and **o**) Rescue experiments using RhoA/ROCKs inhibitors show that LRRC56 regulation of migration and invasion is abrogated in MDA-MB-231 cells. *p < 0.05, **p < 0.01, ***p < *0.001*

To further investigate the complex mechanisms underlying LRRC56’s regulation of RhoA/ROCKs signaling pathway, we reviewed the literature, which indicates that YAP1 modulates this signal, and IFT88 might be one of targets of LRRC56 [7, 37, 38]. We subsequently utilized the ENCORI database to predict potential interactions between LRRC56 and IFT88, which indicated that LRRC56 positively correlated with IFT88 (Fig. 4a). Additionally, data from the GEPIA2 database [39] revealed a significant upregulation of IFT88 in cancer tissues (Fig. 4b). To validate this relationship, co-immunoprecipitation assays confirmed a physical interaction between LRRC56 and IFT88 (Fig. 4c).

The data showed downregulation of LRRC56 led to a corresponding decrease in both IFT88 and YAP1 levels (Fig. 4d-g), suggesting a regulatory link between LRRC56 and these two proteins. To further validate the influence of IFT88 on the YAP1/RhoA/ROCKs pathway, we performed Western blot analyses. Notably, knockdown of IFT88 led to decreased protein expression of YAP1, RhoA, and ROCKs (Fig. 4h-k). To elucidate the underlying mechanisms, we conducted rescue experiments using Fasudil, a potent inhibitor of the RhoA/ROCKs pathway [40]. As shown in Fig. 4l-o, Fasudil effectively counteracted the proliferative, migratory, and invasive effects induced by LRRC56 overexpression in BC cell lines. These results revealed the critical role and interaction between LRRC56 and IFT88 in modulating BC progression via the RhoA/ROCKs pathway.

### Downregulation of IFT88 suppresses breast cancer progression and reverse the effect of LRRC56 in breast cancer

**Fig. 5:**
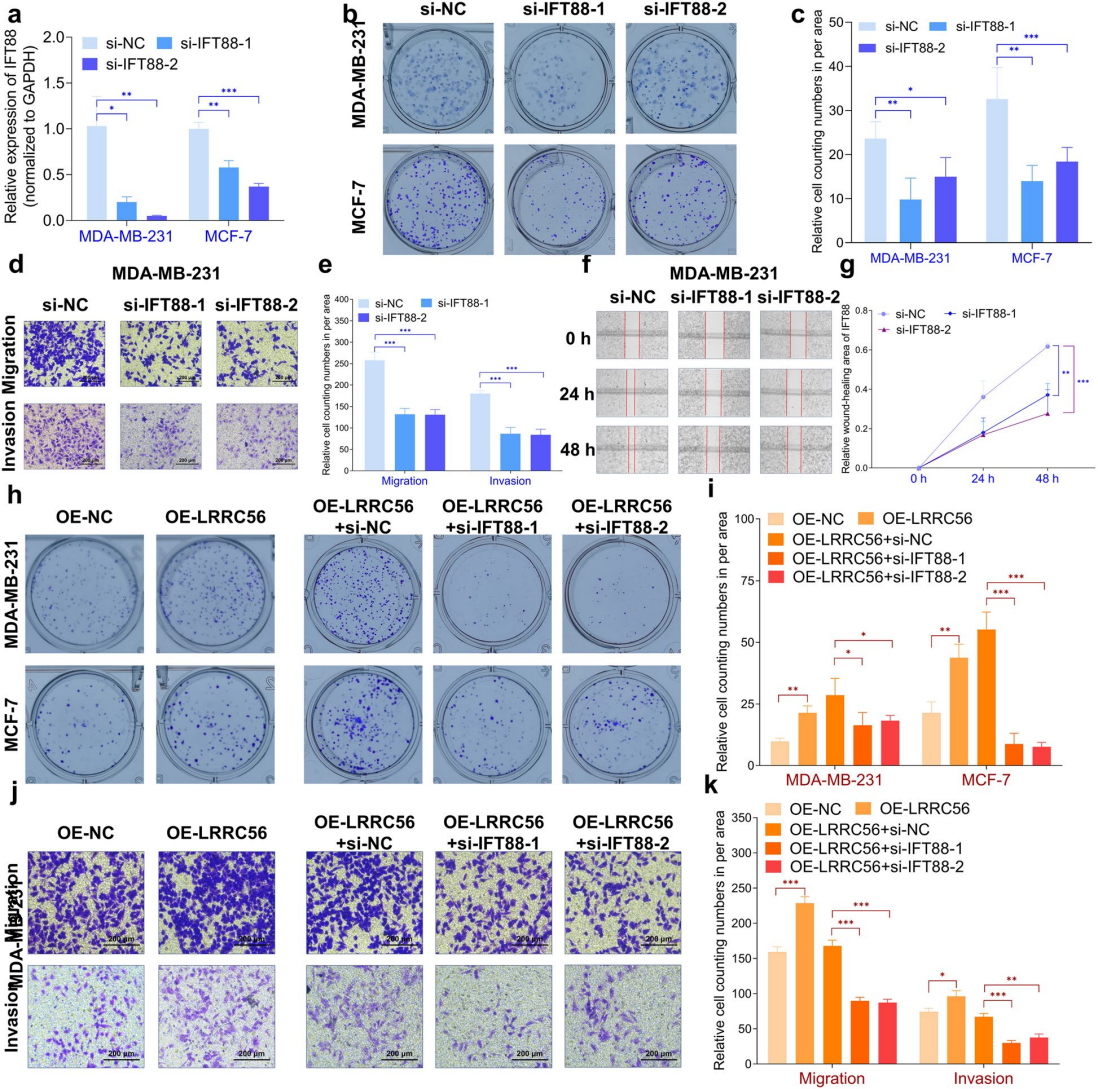
IFT88 regulates BC progression. (**a**)si-IFT88 reduced IFT88 expression in BC cells; (**b **and **c**) Downregulation of IFT88 decreased BC cellular colony formation abilities; (**d **and **e**) Downregulation of IFT88 decreased MDA-MB-231 BC cellular migration and invasion abilities; (**f **and **g**) Downregulation of IFT88 decreased MDA-MB-231 BC cellular wound-healing abilities; (**h **and **i **) Rescue experiments of IFT88 to LRRC56 on colony formation abilities; (**j **and **k**) Rescue experiments of IFT88 to LRRC56 on MDA-MB-231 BC cellular migratory and invasive capacities. *p<0.05, **p<0.01, ***p<0.001

Given the potential interaction between LRRC56 and IFT88, as well as its potential role as an upstream regulator of IFT88, we further investigated the functional significance of IFT88 in BC. To modulate IFT88 expression in BC cells, we designed and synthesized specific siRNAs targeting IFT88 (Fig. 5a). As shown in Fig. 5b-c, upon downregulation of IFT88, the BC cellular proliferative ability decreased (Fig. 5b-c), as well as an inhibition of their migratory capacity (Fig. 5d-g).

To determine whether LRRC56 mediates its biological effects through IFT88, we performed a series of rescue experiments. These experiments included the following conditions: OE-NC (overexpression negative control), OE-LRRC56 (overexpression of LRRC56), OE-LRRC56 + si-NC (overexpression of LRRC56 with non-targeting siRNA control), OE-LRRC56 + si-IFT88-1, and OE-LRRC56 + si-IFT88-2 (overexpression of LRRC56 with two distinct siRNAs targeting IFT88). The experiments indicated that the effects of OE-LRRC56 were reversed upon downregulation of IFT88 (Fig. 5h-k). Collectively, the data demonstrated that IFT88 is crucial for BC progression and functions as a downstream regulator of LRRC56.

### LRRC56 suppresses breast cancer in-vivo progression

**Fig. 6:**
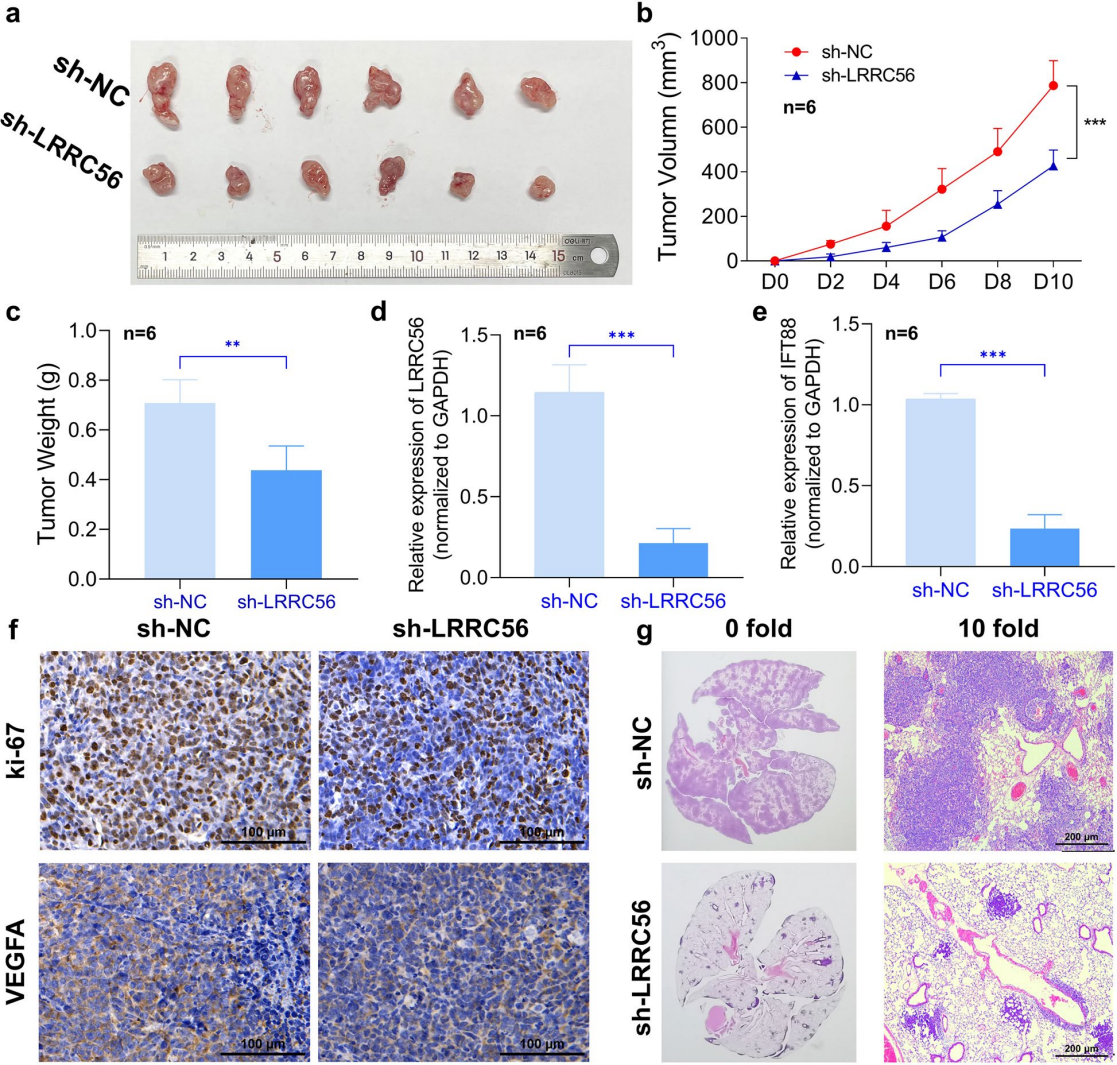
LRRC56 downregulation inhibits BC progression and metastasis in-vivo. (**a**-**b**) Downregulation of LRRC56 inhibited BC in-vivo xenograft proliferation; (**c**) The statistic tumor weight of xenograft (*n* = 6 per group) (**d**) The mRNA expression of LRRC56 in xenograft tissues (*n* = 6 in each group); (**e**) The mRNA expression of IFT88 in xenograft tissues (*n* = 6 per group); (**f**) Downregulation of LRRC56 inhibited ki-67 and VEGFA expression of xenograft; (**g**) Downregulation of LRRC56 inhibited BC lung metastasis in-vivo. *p < 0.05, **p < 0.01, ***p < *0.001*

We generated a plasmid encoding short hairpin RNA (shRNA) to establish a stable MDA-MB-231 cell line with reduced LRRC56 expression. To evaluate the in-vivo function of LRRC56 in BC, the modified cells were then injected into nude mice. The results showed downregulation of LRRC56 significantly impaired xenograft tumor growth (Fig. 6a-c). RNA was extracted from xenograft tumor tissues, followed by RT-qPCR analysis, which revealed reduced expression of both LRRC56 and IFT88 in the sh-LRRC56 tumors (Fig. 6d–e).

Additionally, decreased LRRC56 expression led to lower marker of proliferation Ki-67 (Ki-67) and Vascular endothelial growth factor A (VEGFA) levels in the xenograft tumors (Fig. 6f). To evaluate the effect of LRRC56 in metastasis, a lung metastasis model via tail-vein injection was performed. Mice were divided into two groups: one receiving shRNA targeting LRRC56 (sh-LRRC56) and the other receiving a non-targeting shRNA (sh-NC). Following the injection of 1 million MDA-MB-231 cells per mouse, the development of diffuse pulmonary metastatic lesions was monitored. The sh-LRRC56 group exhibited significantly fewer metastatic lesions (Fig. 6g). Collectively, the data demonstrated LRRC56 promotes BC progression and metastasis in-vivo.

## Discussion

The LRRC protein family, characterized by the presence of a distinctive leucine-rich repeat domain, has attracted increasing attention in cancer research. The LRRC proteins are widely distributed across various cellular compartments, including the cytoplasm, membranes, nucleus, and extracellular matrix. They are implicated in diverse biological functions, including cellular adhesion, polarity determination, intracellular transport, gene expression regulation, and hormone-receptor interactions [41, 42]. Several members, such as LRRC4 [43] and LRRC3B [44], are either absent or significantly downregulated in various malignancies, suggesting their roles as tumor suppressors. In contrast, other members, including LGR4 [45] and LGR5 [46], exhibit heightened expression levels in tumors, underscoring their function as proto-oncogenes. This differential expression pattern underscores the complex and multifaceted roles that LRRC family members play in tumorigenesis and cancer progression. Notably, LRRC15 has been shown to interact with β1-integrin, activating focal adhesion kinase (FAK) signaling and promoting ovarian cancer metastasis [47]. Collectively, the LRRC protein family exhibits diverse involvement in cancer metastasis, providing a conceptual framework for investigating the potential mechanisms and functional roles of LRRC56 in MBC.

Despite increasing interest in LRRC proteins, LRRC56 remains largely understudied, with most prior research focusing on its involvement in development [48], ciliary movement [8, 49], and other related physiological processes. While studies have indicated that LRRC31 can sensitize BMS to radiation therapy by inhibiting DNA repair [50], the function and mechanisms of LRRC56 in BC is still unclear. Nonetheless, through the meticulous analysis of the ONCOMINE database, we observed a significant upregulation of LRRC56 in MBC compared to non-metastatic cases (Supplementary Table 1). The data revealed a potential correlation between high LRRC56 expression and MBC. Based on this observation, we designed and initiated a study to further investigate this hypothesis.

Our functional experiments have shown that knockdown of LRRC56, both in vitro and in-vivo, suppresses the BC cellular proliferative abilities and motility. Given the critical involvement of the RhoA/ROCKs signaling axis in MBC, our previous research has demonstrated that inhibiting this pathway can modulate both the proliferation and motility of BC cells [51, 52]. Building upon these findings, we decided to investigate whether LRRC56 influences BC metastasis through modulation of the RhoA/ROCKs signals. Western blot analysis revealed knockdown of LRRC56 reduced RhoA/ROCKs protein expression, along with decreased phosphorylation of LIM domain kinases (LIMKs) and cofilin (CFL) in both BC cell lines. In contrast, overexpression of LRRC56 enhanced RhoA/ROCKs proteins expression and increased the phosphorylation of LIMKs and CFL (Fig. 3a). To further substantiate the involvement of this signals, we employed Fasudil, a selective RhoA/ROCKs signaling pathway inhibitor, into two BC cell lines overexpressing LRRC56. As shown in Fig. 4l-o, Fasudil effectively inhibited activation the RhoA/ROCKs signals, a thereby neutralizing the proliferative and migratory capabilities induced by LRRC56 overexpression. These findings supported the conclusion that LRRC56 can promote cancer cell proliferation and facilitate BC metastasis by modulating cytoskeletal reorganization, primarily through its regulation of the RhoA/ROCKs signaling pathway.

Numerous studies have demonstrated that RhoA/ROCK signaling is involved in actin polymerization and accelerates F-actin rearrangement, which enables tumor cells to traverse the ECM, thereby increasing the invasive capacity of BC cells [53, 54]. The dynamics of F-actin rearrangement contributed to the EMT process, which facilitates cell detachment and migration [55]. Furthermore, F-actin reorganization impacts focal adhesion turnover, facilitating tumor cell infiltration into surrounding tissues [56]. The actin cytoskeleton also serves as a central interface for interactions with integrins, ECM components, and various signaling pathways mediating cell–cell and cell–ECM communication—processes that are critical for tumor metastasis [57, 58].

To uncover the precise mechanism by which LRRC56 may function in BC, we investigated its potential regulation of the YAP1 protein through its interaction with IFT88, a protein critical for cellular motility [7, 9]. Bioinformatic analysis revealed a positive correlation between LRRC56 and IFT88 expression (Fig. 4a). It has been reported that the RhoA/ROCK pathway upregulates YAP1 transcriptional activity, which mediates cancer invasion [59]. YAP1 is essential for tumor progression [60, 61]. YAP1 has been widely recognized as a key oncogenic driver in BC progression [62, 63] and its dysregulation leads to the activation of numerous pro-proliferative and pro-invasive genes, including CTGF and Cyr61. Simultaneously, YAP1 is also capable of modulating metastasis through its influence on the tumor microenvironment (TME). In BC, elevated expression of YAP1 can activate tumor-associated fibroblasts (CAFs), prompting them to secrete a variety of tumor-promoting factors, including TGF-β and VEGF. These factors, in turn, enhance tumor angiogenesis and ECM remodeling, thereby facilitating the metastasis of BC [63, 64]. Our results data indicated LRRC56 mediated YAP1 expression and potentially to function as an upstream regulator of IFT88, and thereby modulated YAP1, promoting BC metastasis.

IFT88, a key component of the IFT complex, performs a pivotal function in the formation and preservation of cilia [65], influencing cell motility, sensory perception, and development [66, 67]. Beyond its ciliary functions, studies have shown that IFT88 also exerts a cilia-independent regulatory role, particularly in angiogenesis. It regulates microtubule stability and dynamics by interacting with microtubule-binding proteins such as γ-tubulin and microtubule plus-end tracking proteins. This modulation influences the polarization and directed migration of vascular endothelial cells, promoting neovascularization [68]. To evaluate the functional relevance of IFT88, we designed a series of loss-of-function experiments. Our findings demonstrated that downregulation of IFT88 in BC cell lines significantly suppresses BC progression. Western blot analyses further revealed that reduced IFT88 expression led to a corresponding decrease in YAP1 and key proteins involved in the RhoA/ROCKs signaling pathway (Fig. 4h-k). The data indicated that IFT88 functions similarly to oncogenes in BC, promoting malignant behaviors. Furthermore, it exerts an influence on BC metastasis via the RhoA/ROCKs signaling pathway. These findings offer valuable research avenues for investigating the biological function of IFT88 in BC.

Although this work proposed a comprehensive characterization about the LRRC56’s functions in the initiation, progression, and MBC, several limitations remain that warrant further investigation. Firstly, the absence of large-scale validation in clinical samples and insufficient clinical evidence limits our ability to conclusively establish LRRC56 as a key driver of BC metastasis. Additionally, while co-immunoprecipitation confirmed an interaction between LRRC56 and IFT88, our GST pull-down assays indicated that LRRC56 does not directly bind to IFT88 (data not shown), suggesting the involvement of intermediary proteins in this regulatory axis. Furthermore, the precise molecular mechanisms by which LRRC56 regulates IFT88—whether through phosphorylation, ubiquitination, or other post-translational modifications—remain unclear. Addressing such unanswered questions would help to deepen our understanding of BC pathogenesis and develop more effective, targeted therapeutic strategies.

## Conclusion

**Fig. 7:**
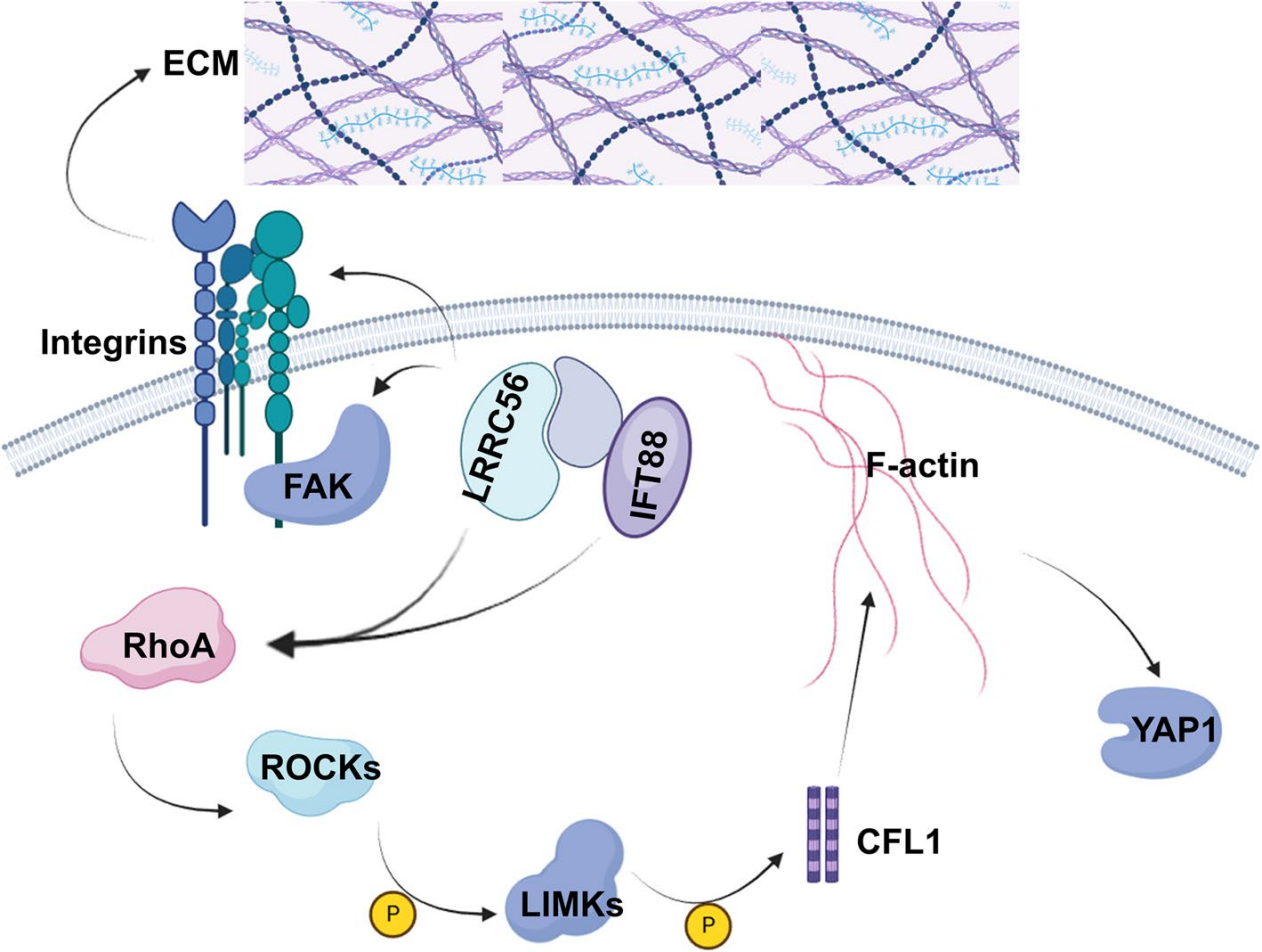
The proposed mechanism of LRRC56 regulation BC progression via RhoA/ROCKs axis. LRRC56 interacts with IFT88 to regulate YAP1 expression via modulating RhoA/ROCKs signaling pathway and regulates integrins and several other key molecules to reprogram ECM and EMT to mediate BC metastasis. Created in BioRender. Fontana. T. (2024) (https://BioRender.com/a23j248)

In summary, our findings preliminarily demonstrate that LRRC56 interacts with IFT88 and modulates the RhoA/ROCK signaling pathway, thereby promoting BC metastasis (Fig. 7). Moreover, further investigation into the mechanistic role of IFT88 in BC may translate these insights into novel therapeutic strategies for MBC.

## Materials and methods

### Breast cancer specimens’ attachment

Specimens of BC were obtained from patients who underwent surgical resection at the Shanghai Tenth People’s Hospital. The tissues were cut into small pieces and cryogenically preserved in liquid nitrogen. Additionally, adjacent non-tumor breast tissues were collected as controls. For accuracy, all samples were histologically confirmed by two independent pathologists. Ethical approval for specimen collection was obtained from the institutional review board of the hospital (Ethic Number: 2021 KN192), and the participants provided written informed consent prior to enrollment.

### RNA extraction and real time quantitative polymerase chain reaction (RT-qPCR)

Total RNA was isolated from BC cells using an RNA Isolation Kit (Beyotime, Shanghai, China), followed by reverse transcription into complementary DNA (cDNA) utilizing the HiScript III 1 st Strand cDNA Synthesis Kit with gDNA wiper (Vazyme, Nanjing, China), in accordance with the manufacturer’s instructions. RT-qPCR was subsequently performed. GAPDH served as an internal control for normalization of gene expression, and the relative mRNA levels were determined using the 2^−ΔΔCt^ method [69].

### Cell culture, transfection and stable cell line establishment

Both BC cell lines were sourced from a research institute affiliated with the Chinese Academy of Sciences, located in Shanghai, China. Cells were cultured in in Dulbecco’s Modified Eagle’s Medium (DMEM, WISENT, Canada), supplemented with 10% fetal bovine serum (FBS, absin, Shanghai, China) and 1% penicillin–streptomycin (PS, Beyotime, Shanghai, China).

Small interfering RNAs (siRNAs) and plasmids were custom-designed and synthesized by Generay Biotech, Shanghai, China. For the purpose of transient transfection, cells were inoculated into six-well culture plates, with each well receiving approximately 2 × 10^5^ cells for siRNAs or 2.5 × 10^5^ cells per well for plasmids. Then, Lipofectamine 8000 (Lipo8000; Beyotime, Shanghai, China) was employed to transfect the cells, following the supplier’s guidelines without deviation.

Additionally, GentleGen (Suzhou, China) designed and synthesized the short hairpin RNA (shRNA) plasmids. For establishing stable cell lines, transduction was performed using the LentiFit transfection kit (Hanbio, Shanghai, China) in strict accordance with the supplier’s protocol. Following infection, puromycin (4 μg/mL; Beyotime, Shanghai, China) was applied to selectively eliminate untransfected cells.

### Proliferation assays

Following transfection, BC cells were resuspended and transferred into 96-well culture plates to carry out the 3-(4,5-dimethylthiazol-2-yl)−2,5-diphenyltetrazolium bromide (MTT) assay (Beyotime, Shanghai, China). Each well was seeded with 2 × 10^3^ cells. Measurement absorbance at 490 nm optical density was used to quantify the extent of cellular proliferation, which was performed once every 24 h over a period of five days. Additionally, for the colony formation assay, cells were plated into 12-well plates and incubated under standard culture conditions to allow colony development with 800 cells per well. Following 7–10 days of incubation, cells were treated with 4% paraformaldehyde (PFA; Servicebio, Wuhan, China) to achieve fixation, and then visualized by staining with 0.1% crystal violet solution (Yeasen, Shanghai, China).

### Wound healing assay

Transfected cells were transferred and cultured in 12-well plates. When the BC cell monolayers in each well achieved approximately 90% confluency, a uniform scratch was generated across the adherent cell layer by gently dragging a sterile 200 μl pipette tip over the surface to mimic a wound. Subsequently, the culture medium was replaced with 2% FBS DMEM. Images of each wound were captured immediately (0 h), and then again at 24 h and 48 h post-medium change. Following this, the migration speed of the cells was quantitatively assessed by measuring the change in the width of the scratches over time.

### Transwell assay

Transfected BC cells were prepared as single-cell suspensions in DMEM and a volume of 200 μl of the single-cell suspension was carefully added to the upper chamber of each 24-well transwell insert (Corning, NY, USA). Cell migratory and invasive behaviors were assessed in the absence or presence of a Matrigel coating (Yeasen, Shanghai, China), respectively. Following a 16-h incubation period, the lower surfaces of the inserts were gently rinsed with phosphate-buffered saline (PBS, Servicebio, Wuhan, China) to remove non-migrated cells. The cells were then fixed using 4% PFA and stained with 0.1% crystal violet. After air-drying, images of the bottoms were captured for analysis. The quantity of transwell cells was counted via representative area and the experiments were conducted for three times.

### 5-Ethynyl-2′-deoxyuridine (EdU) assay

The EdU assay was conducted using the BeyoClick™ EdU Cell Proliferation Kit with Alexa Fluor 594 (Beyotime, Shanghai, China). Cells were first washed with PBS, followed by incubation in EdU solution for two hours. Following this, cells were treated with Hoechst solution to visualize nuclear morphology. The samples were then subjected to additional washing and visualized using an inverted fluorescence microscope (Nikon Ti2-Elements, Japan). Quantification of EdU-positive cells was performed using Image J software (version 1.46; National Institutes of Health, Bethesda, MD, USA).

### Protein extraction

The BC cells, following treatment, were retrieved from the incubator and subjected to three consecutive washes using pre-chilled PBS. Subsequently, cell lysis was carried out using RIPA buffer (Beyotime, Shanghai, China) containing 1 mM phenylmethylsulfonyl fluoride (PMSF, Beyotime, Shanghai, China) and a 1 mM protease inhibitor cocktail (InStab™ Phosphatase Inhibitor Cocktail 1 (100 × in DMSO), Yeasen, Shanghai, China) to prevent protein degradation. Total protein content in the lysates was then quantified using a BCA assay kit (Epizyme, Shanghai, China), following the manufacturer’s protocol.

### Western Blot (WB)

Each sample (40 μg) was subjected to SDS–polyacrylamide gel electrophoresis (Beyotime, Shanghai, China) with a gradient density ranging from 6 to 12%. Upon completion of electrophoretic separation, protein samples were transferred onto 0.45 μm nitrocellulose (NC) membranes (Cytiva, Massachusetts, USA) using electroblotting. To minimize non-specific binding, the membranes were first blocked using a suitable blocking buffer. Thereafter, they were sectioned based on the anticipated molecular weights and incubated with target-specific primary antibodies. Subsequently, incubation with appropriate secondary antibodies was carried out at room temperature for an hour. Membranes were rinsed three times using Tris-buffered saline with 0.1% Tween (TBST, Epizyme, Shanghai, China) to eliminate any unbound antibody residues. Finally, protein bands were visualized using the Odyssey imaging system (LI-COR Biosciences, Lincoln, NE, USA) in combination with the Enhanced ECL Chemiluminescent Substrate Kit (36222ES60, Yeasen, Shanghai, China). The specific primary antibodies information is detailed in Supplementary data 1.

### Co-immunoprecipitation (co-IP)

BC cells were lysed using an IP-compatible lysis buffer (Beyotime, Shanghai, China). For each reaction, an aliquot of 1000 μg total protein was first collected from each sample. The proteins were then resuspended in 500 μl of IP lysis buffer for subsequent antibody incubation. Subsequently, the Co-IP kit (abs955, Absin, Shanghai, China) was used to perform the experiment. Finally, WB analysis was performed to detect and identify the bound proteins present in these samples.

### In-vivo xenograft and lung-metastasis experiments

We conducted in-vivo experiments utilizing genetically modified MDA-MB-231 cells with LRRC56 knocked out (KO), in parallel with their corresponding negative controls as previously described. Female nude mice aged 4 to 6 weeks (JiHui, Shanghai, China) were housed under specific pathogen-free (SPF) conditions. This endeavor was granted ethical approval by the institutional Animal Ethics Committee (Ethic No.: SHDSYY-2021–4531), Shanghai Tenth People’s Hospital. Prior to the xenograft experiments, MDA-MB-231 cells were propagated in standard 10 cm dishes and suspended in pre-chilled PBS (Servicebio, Wuhan, China). To establish the tumor xenograft model, each mouse received a subcutaneous injection of 100 μl cell suspension, containing 1 × 10^6^ MDA-MB-231 cells, into the axillary area. Body weight and tumor dimensions were assessed every other day throughout the experiment. Tumor volume was determined according to the calculation method described in our earlier publication [69]. Upon reaching a tumor volume nearing 1 cm^3^ in either group, we concluded the xenograft experiment. The tumors were excised subcutaneously, with portions being preserved in 4% PFA for fixation and others rapidly frozen for analysis. To evaluate pulmonary metastasis, a 100 μl suspension containing 5 × 10^5^ cells was rapidly administered via intravenous injection through the tail vein of each mouse. The mice were weighed bi-daily, and the lung metastasis experiment was terminated upon significant weight loss or dyspnea observed in either group. Following this, the thoracic cavity was meticulously dissected to procure the lungs and ribs, which were then fixed for further analysis.

### Hematoxylin–Eosin (HE) and Immuno-Histochemical (IHC) Staining

The paraffin-embedded tumors and tissues underwent a process of sectioning, with the assistance of Servicebio (Wuhan, China), for subsequent HE staining and IHC staining. The antibodies utilized in the IHC staining procedure were as follows: Ki-67 (1:300, Servicebio, Wuhan, China; Cat# GB121141) and VEGF (1:200, Servicebio, Wuhan, China; Cat# GB14165).

### Statistical analysis

All statistical analyses were conducted using GraphPad Prism software (version 5.0, San Diego, CA, USA). Each dataset included triplicate measurements obtained from at least three independent experiments. Results were expressed as the mean with corresponding standard deviations (mean ± SD) to ensure data reliability. Statistical comparisons were carried out using either an unpaired Student’s t-test or two-way ANOVA, and a *p*-value < 0.05 was considered statistically significant. Biologically, all experiments were conducted in triplicate to ensure reproducibility and reliability of the results.

## Supplementary Information

Supplement Figure 1: The Quantification of Western blot analysis for RhoA/ROCKs pathway, ECM, and EMT protein expression. (a and b) Quantitative analysis of RhoA/ROCKs pathway proteins in MDA-MB-231 cells; (c and d) Quantitative analysis of RhoA/ROCKs pathway proteins in MCF-7 cells;(e and f) Quantitative analysis of ECM-related proteins in MDA-MB-231 cells;(g and h) Quantitative analysis of ECM-related proteins in MCF-7 cells;(i and j) Quantitative analysis of EMT markers in MDA-MB-231 cells;(k and l) Quantitative analysis of EMT markers in MCF-7 cells. ns, not statistically significant; *p < 0.05, **p < 0.01, ***p < 0.001.

Supplementary Material 2.

Supplementary Material 3.

## Data Availability

All data used in our study can be acquired from the cancer ENCORI repository (https://starbase.sysu.edu.cn/panCancer.php), the bc-GenExMiner (https://bcgenex.ico.unicancer.fr/BC-GEM/GEM-Accueil.php?js=1), and the GEPIA2 database(http://gepia2.cancer-pku.cn/#index). Any other data are available from the corresponding author on reasonable request. Research mechanism diagram was Created in BioRender. Fontana. T. (2024) https://BioRender.com/a23j248. Software and resources used for analysis and plotting are described in each method section.

## Abbreviations

BC: Breast Cancer
BSA: Bovine Serum Albumin
CAFs: Cancer-Associated Fibroblasts
CFL1: Cofflin1
co-IP: Co-Immunoprecipitation
DAPI: 4',6-Diamidino-2-phenylindole
DMEM: Dulbecco’s Modified Eagle’s Medium
ECM: Extracellular Matrix
EdU: 5-Ethynyl-2′-deoxyuridine
EMT: Epithelial-Mesenchymal Transition
FAK: Focal Adhesion Kinase
FBS: Fetal Bovine Serum
HE: Hematoxylin-Eosin
IFT: Intraflagellar Transport
IFT88: Intraflagellar Transport 88
IHC: Immunohistochemical
Ki-67: Marker of proliferation Ki-67
KO: Knocked Out
LIMKs: LIM domain kinases
LRRC: Leucine-Rich Repeat-Containing
LRRC56: Leucine-Rich Repeat-Containing 56
MBC: Metastasis Breast Cancer
MTT: 3-(4,5-Dimethylthiazol-2-yl)-2,5-diphenyltetrazolium bromide
NC: Nitrocellulose
ODAs: Outer Dynein Arms
PBS: Phosphate Buffered Saline
PFA: Paraformaldehyde
PMSF: Phenylmethylsulfonyl Fluoride
PS: Penicillin-Streptomycin
RhoA: Ras homolog family member A
ROCKs: Rho-associated protein kinases
RT-qPCR: Real-Time quantitative Polymerase Chain Reaction
shRNA: Short hairpin RNA
siRNAs: Small interfering RNAs
SPF: Specific Pathogen-Free
TBS: Tris-Buffered Saline
VEGFA: Vascular endothelial growth factor A
YAP1: Yes-associated protein 1
