# Supplementary figures and images for: Leucine-rich repeat-containing 56 promotes breast cancer progression via modulation of the RhoA/ROCKs signaling axis
Source: Mol Biomed. 2025 May 19;6:31. doi: 10.1186/s43556-025-00271-w (PMC12089637; doi:10.1186/s43556-025-00271-w)

MDA-MB-231

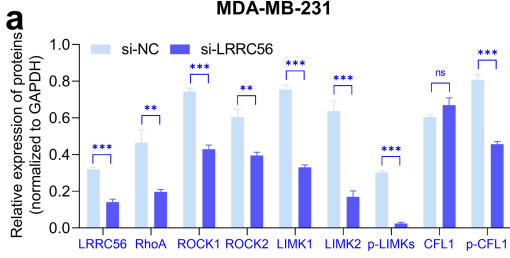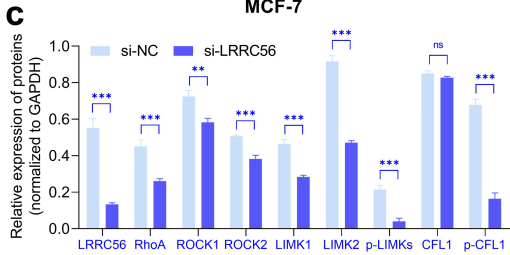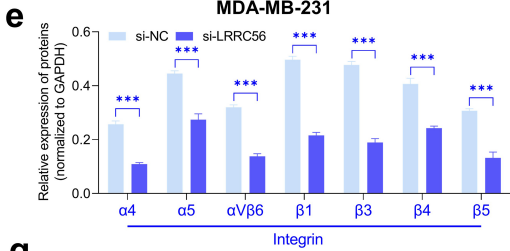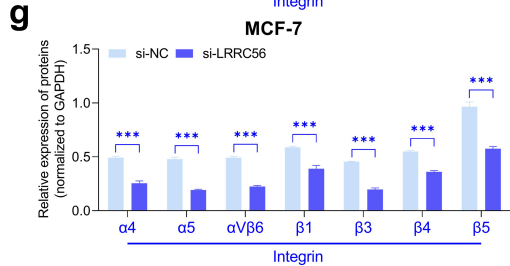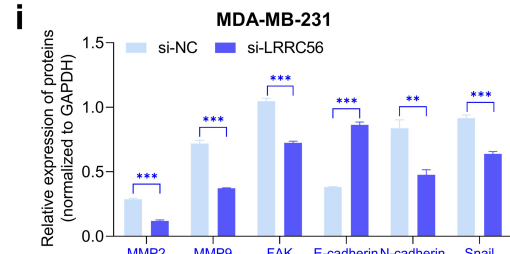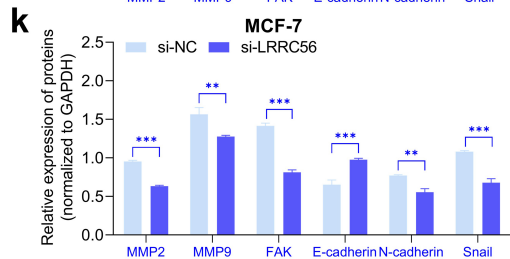

MDA-MB-231

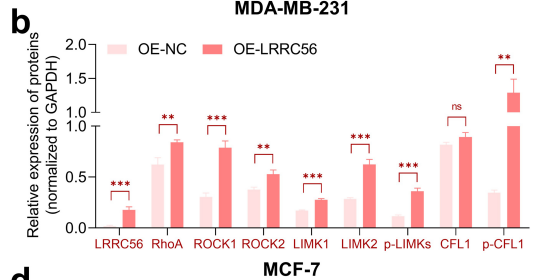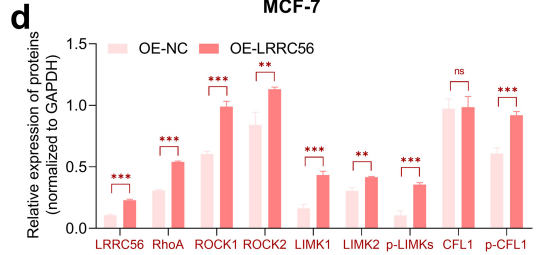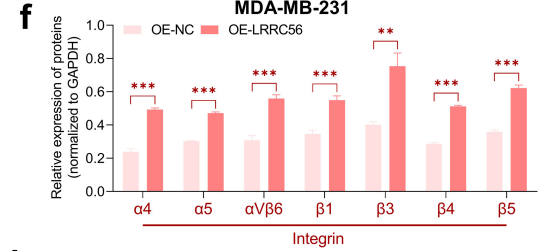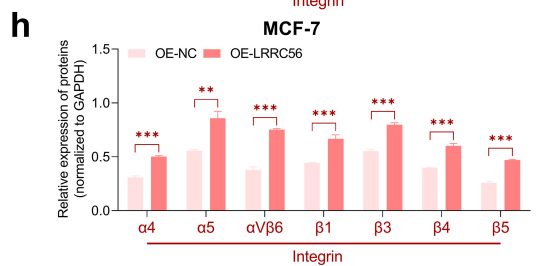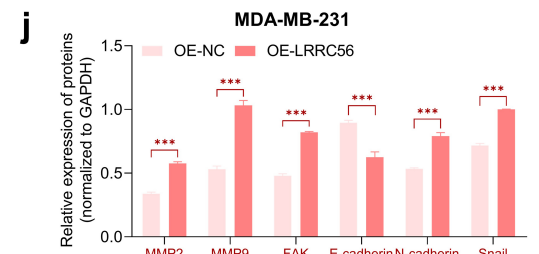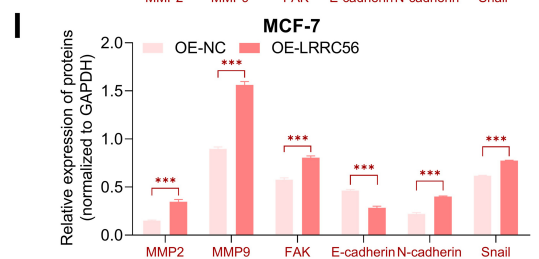

Supplement: Supplementary file 1 — Supplement Figure 1: The Quantification of Western blot analysis for RhoA/ROCKs pathway, ECM, and EMT protein expression. (a and b) Quantitative analysis of RhoA/ROCKs pathway proteins in MDA-MB-231 cells; (c and d) Quantitative analysis of RhoA/ROCKs pathway proteins in MCF-7 cells;(e and f) Quantitative analysis of ECM-related proteins in MDA-MB-231 cells;(g and h) Quantitative analysis of ECM-related proteins in MCF-7 cells;(i and j) Quantitative analysis of EMT markers in MDA-MB-231 cells;(k and l) Quantitative analysis of EMT markers in MCF-7 cells. ns, not statistically significant; *p < 0.05, **p < 0.01, ***p < 0.001. [file 43556_2025_271_MOESM1_ESM.pdf]
