# Supplementary material for: Leucine-rich repeat-containing 56 promotes breast cancer progression via modulation of the RhoA/ROCKs signaling axis
Source: Mol Biomed. 2025 May 19;6:31. doi: 10.1186/s43556-025-00271-w (PMC12089637; doi:10.1186/s43556-025-00271-w)
Supplement: Supplementary file 3 — Supplementary Material 3. [file 43556_2025_271_MOESM3_ESM.pdf]

Leucine-rich repeat-containing 56 promotes breast cancer progression via modulation of the RhoA/ROCKs signaling axis

Xiqian Zhou[1 #], Jiaxin Wang [1,2#], Meiling Lu[3], Lin Fang[1], Junyong Zhao[1 *], Dengfeng Li [1*]

1 Department of Breast and Thyroid Surgery, Shanghai Tenth People's Hospital, Institute of Breast Disease, Tongji University School of Medicine, Shanghai, 200072, China.

2 School of Medicine, Tongji University, Shanghai, 200092, China

3 Department of Central Laboratory, Shanghai Tenth People's Hospital of Tongji University, School of Life Sciences and Technology, Tongji University, Shanghai, 200072, China.

# Equal contribution

* Co-corresponding authors

Dengfeng Li, MD, PhD, Department of Breast and Thyroid Surgery, Shanghai Tenth People's Hospital, Institute of Breast Disease, Tongji University School of Medicine, NO.301 Yanchang Middle Road, Shanghai 200072, People's Republic of China. Tel: 021-66301057. Email: 711ldf@tongji.edu.cn

Junyong Zhao, MD, Department of Breast and Thyroid Surgery, Shanghai Tenth People's Hospital, Institute of Breast Disease, Tongji University School of Medicine, NO.301 Yanchang Middle Road, Shanghai 200072, People's Republic of China. Tel: 021-66301057. Email: 1610705@tongji.edu.cn

Supplement data 1

1.The sequences of primers were listed as below:

**Name**		**Primer (5'- 3')**
LRRC56	Forward	GCCATCAAGAAGGGCAACG
	Reverse	AGCTCGGGGTCAAGTCTCC
IFT88	Forward	TCCTGAAACTTCACGCAATCC
	Reverse	GACCACCTGCATTAGCCATTC
GAPDH	Forward	GGAAGCTTGTCATCAATGGAAATC
	Reverse	TGATGACCCTTTTGGCTCCC

2.The sequences of siRNAs were listed as below:

**Name**		**Sequence (5'- 3')**
Homo-LRRC56-904	Sense	CCUCUUUGCCAGCACUUAATT
	Antisense	UUAAGUGCUGGCAAAGAGGTT
Homo-LRRC56-915	Sense	GCACUUAAGGAACUCUACGTT
	Antisense	CGUAGAGUUCCUUAAGUGCTT
HOMO-IFT88-319	Sense	CAAGACAUCUCUGGCAUCAUCAAUA
	Antisense	UAUUGAUGAUGCCAGAGAUGUCUUG
HOMO-IFT88-418	Sense	CAAAGCAGCUUUGAGAGGCUCUGCA
	Antisense	UGCAGAGCCUCUCAAAGCUGCUUUG
siRNA-negative control	Sense	UUCUCCGAACGUGUCACGUTT
	Antisense	ACGUGAACGUUCGGAGAATT

- The vector of over-expression plasmids were pLV3-CMV-3xFLAG-copGFP-Puro (MiaoLing Biology, Wuhan, China).
- the vectors of shRNAs were PLKO.1 (GentleGen, Suzhou, China).

3. The primary and secondary antibodies involved were listed as followings:

**Name**	**Dilution ratio**	**Cat#**	**Company**
LRRC56	1:500	NBP1-70026	NOVUS, Colorado, USA

Name	Dilution ratio	Cat#	Company
RhoA	1:1000	2117T	Cell signaling Technology, Massachusetts, USA
ROCK1	1:1000	4035S	Cell signaling Technology, Massachusetts, USA
ROCK2	1:500	21645-1-AP	Proteintech, Wuhan, China
LIMK1	1:1000	3842S	Cell signaling Technology, Massachusetts, USA
LIMK2	1:1000	3845T	Cell signaling Technology, Massachusetts, USA
p-LIMKs	1:1000	3841T	Cell signaling Technology, Massachusetts, USA
CFL1	1:500	ARG51149	Arigo Biolaboratories, Bio-platform, Shanghai, China
P-CFL1	1:500	ARG20528	Arigo Biolaboratories, Bio-platform, Shanghai, China
GAPDH	1:1000	gb15004-100	Servicebio, Wuhan, China
Integrin α4	1:1000	A4054	Abclonal, Wuhan, China
Integrin α5	1:1000	A22706	Abclonal, Wuhan, China
Integrin αvβ6	1:1000	bs-5791R	Bioss, Beijing, China
Integrin β1	1:1000	A23497	Abclonal, Wuhan, China
Integrin β3	1:1000	A19073	Abclonal, Wuhan, China
Integrin β4	1:500	A4596	Abclonal, Wuhan, China
Integrin β5	1:1000	A23428	Abclonal, Wuhan, China
MMP2	1:1000	66366-1-Ig	Proteintech, Wuhan, China
MMP9	1:500	10375-2-AP	Proteintech, Wuhan, China
FAK	1:5000	66258-1-Ig	Proteintech, Wuhan, China
E-cadherin	1:20000	20874-1-AP	Proteintech, Wuhan, China
N-cadherin	1:1000	A19083	Abclonal, Wuhan, China
Snail	1:500	A24806	Abclonal, Wuhan, China
YAP	1:1000	A1002	Abclonal, Wuhan, China
IFT88	1:1000	13967-1-AP	Proteintech, Wuhan, China
Recombinant Anti-GST Tag antibody	1:1000	GB15085-100	Servicebio, Wuhan, China
Rabbit pAb Control IgG	3μg (for co-IP)	AC005	Abclonal, Wuhan, China
Goat Anti-Rabbit IgG (Dylight 800)	1:2000	A23920	abbkine, California, USA
Goat Anti-Mouse IgG (Dylight 800)	1:2000	A23910	abbkine, California, USA
HRP-conjugated Goat anti-Rabbit IgG (H+L)	1:4000	AS014	Abclonal, Wuhan, China

Name	Dilution ratio	Cat#	Company
HRP-conjugated Goat anti-Mouse IgG (H+L)	1:4000	AS003	Abclonal, Wuhan, China

4. The antibodies applied in IHC staining are listed as follows:

Name	Dilution ratio	Cat#	Company
Ki-67	1:300	GB121141	Servicebio, Wuhan, China
VEGF	1:200	GB14165	Servicebio, Wuhan, China
